# 27. Failure to stand corrected: camptocormia, a rare cause of bent spine

**DOI:** 10.1093/rap/rky033.018

**Published:** 2018-09-20

**Authors:** Yik Long Man, Michael Rose, Gerald Coakley

**Affiliations:** 1 Rheumatology, Queen Elizabeth Hospital, London, UNITED KINGDOM; 2 Neurology, Queen Elizabeth Hospital, London, UNITED KINGDOM

**Introduction:** Rheumatologists are frequently referred elderly patients with back pain and increased thoracic kyphosis. This is usually attributable to vertebral fractures secondary to osteoporosis. We present a case of camptocormia, a rare cause of bent spine where there is increased forward flexion of the thoracolumbar spine secondary to weakness of the extensor paravertebral muscles. Strikingly, there is complete resolution of this deformity on lying supine.

**Case description:** An 85 year old former dancer was referred to the rheumatology clinic with a worsening stooped posture which developed over one year. Her GP was concerned that she may have had osteoporotic vertebral fractures. Her stoop was so severe that she could not look straight ahead and it had a major impact on her activities of daily living. She had mild thoracic back pain. She reported no neurological symptoms. On examination, she had a bent spine with marked kyphosis. Of note, there was complete resolution of the spinal deformity when she lay supine on the examination couch. She had a normal neurological examination of the upper and lower limbs, although there was notable head titubation. Investigations included plain radiographs and an MRI spine. These showed degenerative changes and minor spinal canal stenosis at several levels, but no evidence of any vertebral fractures to explain the bent spine. A diagnosis of camptocormia was proposed and she was referred to neurology for further investigations. The neurology opinion was that she had camptocormia secondary to dystonia in view of a stiff neck and head titubation, and she was given co-careldopa for symptom management. An MRI head was also organised which showed subtle, non-specific T2 changes within the basal ganglia and thalami bilaterally, which would most likely represent small vessel ischaemia.

**Discussion:** Camptocormia is also known as bent spine syndrome; the characteristic feature is severe forward flexion of the thoracolumbar spine which is present on walking or standing with complete resolution in the supine position. It is caused by weakness in the extensor paravertebral muscles. Vertebral fractures secondary to osteoporosis are commonly encountered in the rheumatology clinic, presenting as back pain and increased thoracic kyphosis. This is usually confirmed on imaging. However, in the absence of any vertebral fractures on imaging, it is necessary to consider alternative diagnoses. Furthermore, if there is resolution of the bent spine on lying supine, then a diagnosis of camptocormia should be considered. There are many causes of camptocormia, but the most recognised association is with Parkinson’s disease. It typically occurs in patients with more severe Parkinson’s disease. Other central nervous system disorders associated with camptocormia include dystonia, multisystem atrophy and Alzheimer’s disease. It can also be associated with disorders of the peripheral nervous system including myasthenia gravis and chronic inflammatory demyelinating polyneuropathy. Interestingly, it can be caused by psychiatric disorders in rare cases, and it is well recognised as a conversion disorder in traumatised military personnel. The diagnosis is based on clinical findings on examination, with the support of imaging, electromyography or muscle biopsy to identify the underlying cause of camptocormia. The management is therefore directed towards treating the underlying disease which would usually involve onward referral to the Neurology team and general measures including physiotherapy and orthoses.

**Key Learning Points:** This case highlights the importance of considering neurological disorders when assessing a patient with musculoskeletal symptoms. In the absence of vertebral fractures, consider camptocormia as a cause of bent spine and check whether this improves with lying supine on the examination couch. It is also important to look for any other neurological signs that would point towards the underlying diagnosis, as managing the underlying diagnosis is key to treating camptocormia and reducing morbidity.

**Disclosure: Y. Man:** None. **M. Rose:** None. **G. Coakley:** None.

